# Settling moths are the vital component of pollination in Himalayan ecosystem of North-East India, pollen transfer network approach revealed

**DOI:** 10.1038/s41598-022-06635-4

**Published:** 2022-02-17

**Authors:** Navneet Singh, Rajesh Lenka, Pallab Chatterjee, Dipayan Mitra

**Affiliations:** 1 Zoological Survey of India, New Alipore, Kolkata, 700053 India; 2 Department of Zoology, Ballygunge Science College, University of Calcutta, Kolkata, India

**Keywords:** Ecology, Zoology

## Abstract

Majority of the pollination related studies are based on the diurnal pollinators, and the nocturnal pollinators received less scientific attention. We reveal the significance of settling moths in pollination of angiosperm families in Himalayan ecosystem of North-East India. The refined and novel method of pollen extraction from the proboscides provides a more robust assessment of the pollen carrying capacity. The study is based on one of the largest data sets (140 pollen transporter moth species (PTMS)), with interpretation based on seasonal as well as altitudinal data. In the present study about 65% moths (91 species) carried sufficient quantities of pollen grains to be considered as potential pollinators (PPMS). *Teliphasa* sp. (Crambidae) and *Cuculia* sp. (Noctuidae) are found to carry the highest quantity of pollen. We found pollen grains of 21 plant families and the abundant pollen are from Betulaceae, Fabaceae, Rosaceae and Ericaceae. Species composition of PTMS and PPMS in pre-monsoon, monsoon, and post-monsoon revealed the dominance of Geometridae. Maximum diversity of PTMS and PPMS is found from 2000 to 2500 m altitude. The nocturnal pollen transfer network matrices exhibited high degree of selectivity (H_2_ʹ = 0.86).

## Introduction

Pollination is an indispensable ecological process for the continuity of germplasm^1^ and is mainly driven by wind, water and animals as vectors^1,2^. About 87.5% of all angiosperms are mainly pollinated by animals^3–7^. Among these, pollinating insects have important mutualistic relationships with angiosperms, which are essential for the conservation of wild as well as agricultural landscapes^2,8^.

So far, most research on pollinating insects has focused on diurnal pollinators, and the nocturnal pollinators have traditionally received less scientific attention^9^. Despite recent research in the last many years on their role in pollination, many questions left unanswered. An important review by Macgregor and co-workers revealed that 289 species of plants (mostly angiosperms from the orders Caryophyllales, Ericales, Gentianales, and Lamiales) under 75 families are partially or entirely pollinated by 21 moth families^8^. Some limited studies related to the comparable importance of the moths as pollinators to that of diurnal pollinators^10–12^, advocate the benefits of moths over the diurnal pollinators through mechanisms such as (1) longer-distance dispersal of pollen^13,14^, (2) good seed set despite deposition of fewer pollinia^15–17^ and (3) increased pollination efficiency due to pollen deposition in a single visit^18^.

Among the moths, the settling moths (mainly of families other than Sphingidae, which hover when feeding from flowers) are extremely common and diverse flower visitors^19^ but are less studied for their role in pollination. Many studies have focused on pollination by Sphingidae (i.e. the Sphingophily)^20–31^. The role of settling moths as pollinators i.e. the Phalaenophily^29^ has also been studied, but arguably to a lesser degree^19,27^. Settling moths may potentially be effective pollinators of generalist plants which are not visited by hawkmoths or where the hawkmoths are not common^32^.

The noteworthy studies related to pollination by settling moths are sporadic, few and limited to certain regions of the world viz. in eastern England^12^, East Asia^33,34^, North Europe and America^35,36^. Other studies conducted on the pollination of plant families, Asteraceae, Ericaceae, Plantaginaceae, Myrtaceae, Campanulaceae, Thymelaeaceae, revealed the participation of settling moths^12,27,37,38^.

The Indian moths are negligibly studied for their role in pollination and only four papers could be reviewed for this aspect. Paul^39^ studied the pollination efficiency of noctuid moth species in urban areas of Delhi. Sarkar and Sreedevi^40^ studied the nocturnal insect pollinators of bottle gourd and ridge gourd crop of Andhra Pradesh and reported three species of settling moths, *Arthroschista hilaralis* (Crambidae), *Diaphania indica* (Crambidae) and *Anadevidias peponis* (Noctuidae), as major pollinators. The seasonal dynamics of plant-pollinator networks in agricultural landscapes of West Bengal and the pollination of medicinal plants in Tripura revealed the participation of settling moths^41,42^. The aim of the present study is to investigate the role of settling moths in pollination of angiosperm families distributed in Himalayan ecosystem of North-East India by quantifying various network level indices along with the effect of various seasons and altitudinal gradient on the pollen carrying capacity. The pollen transport does measure pollination success, but is a proxy whereby we can begin to assess involvement in pollination process^43^. The present study is based on the dataset generated from the light trapping at 24 field sites in Himalayan ecosystem of North-East India, over the period of 13 months.

## Results

### Settling moths as pollen vectors

**Figure 1:**
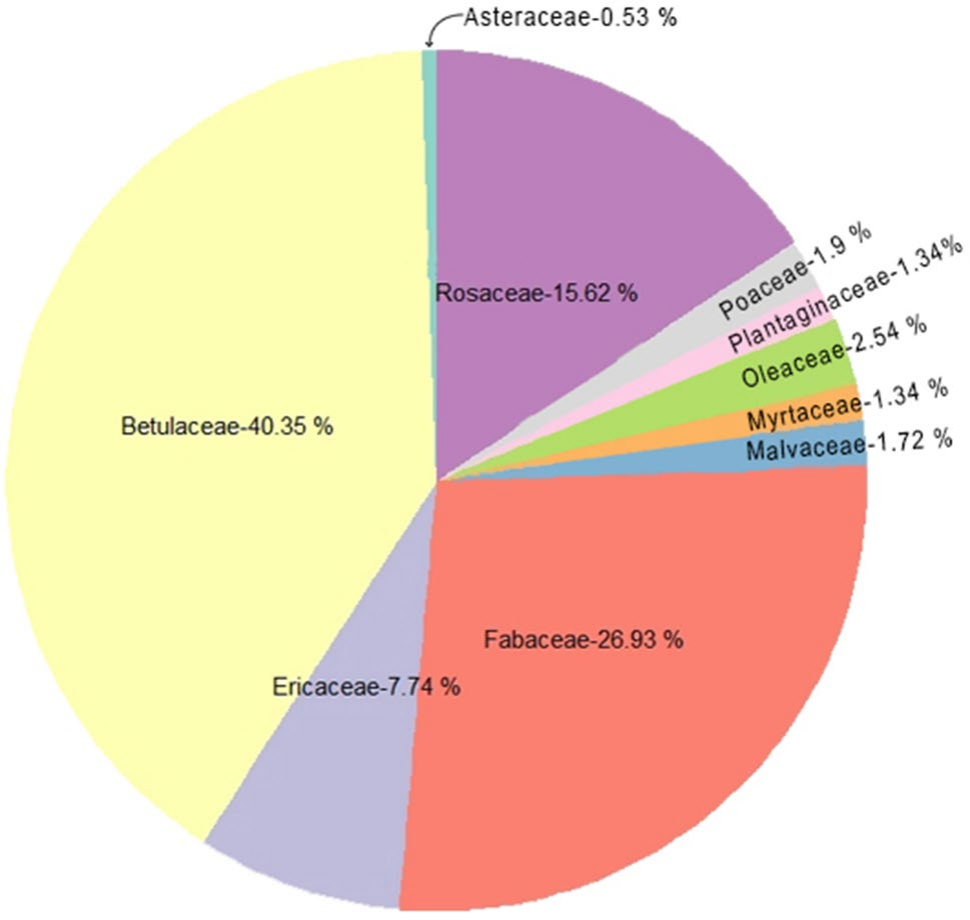
Pie-chart of ten major plant families and their pollen load percentage found on proboscides of PTMS.

**Figure 2:**
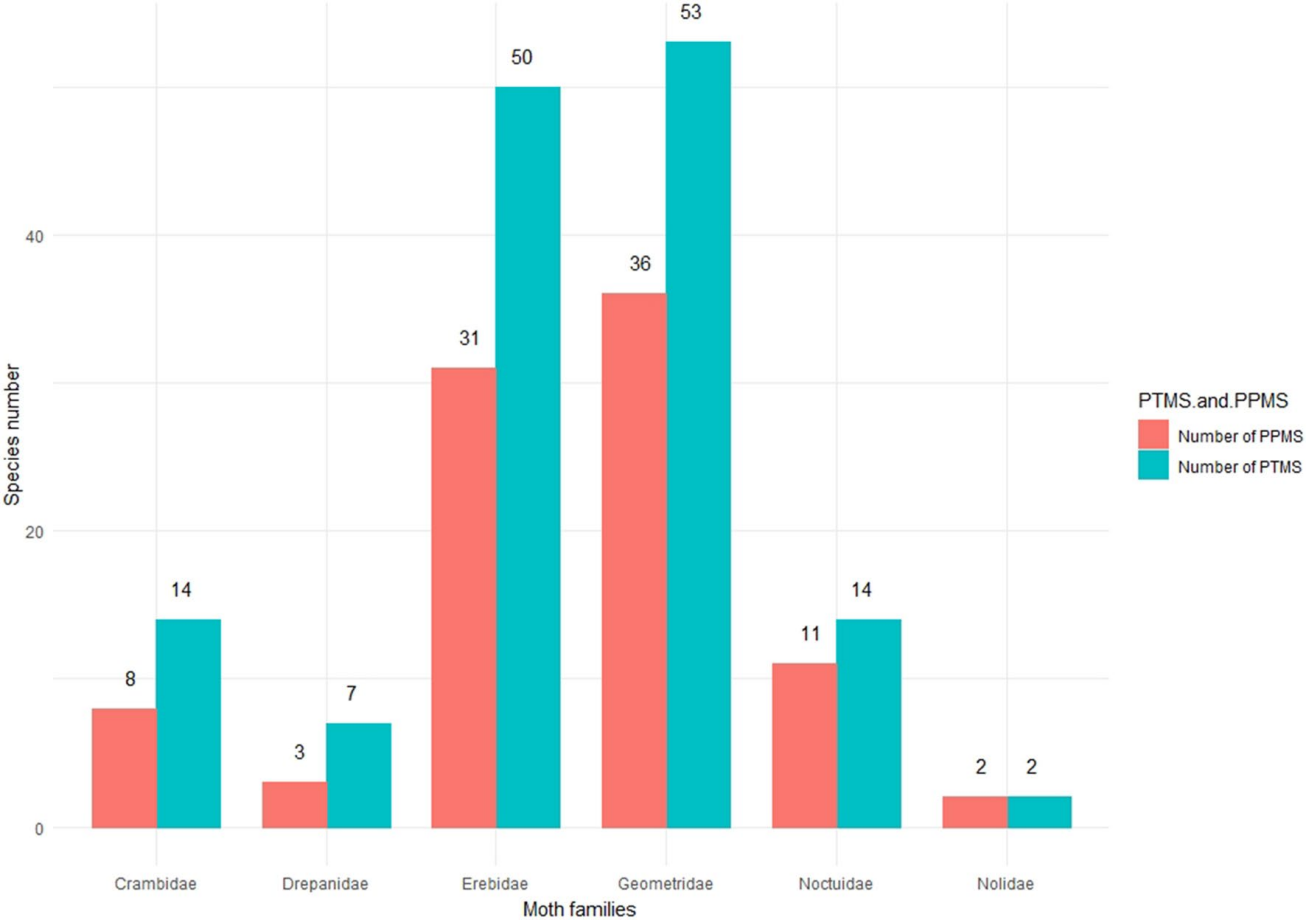
Family-wise species composition of PTMS and PPMS.

We identified 140 moth species in 18 subfamilies and 6 families of settling moths carrying pollen grains on their proboscis, and termed them as ‘pollen transporter moth species’ (PTMS). Out of the 140 PTMS, the proboscides of 91 moth species are found to carry five or more than five pollen grains of one or another plant family. Herein, we termed the 91 moth species as ‘potential pollinator moth species’ (PPMS) by following Macgregor et al.^8^ and Devoto et al.^44^ (Figs. 1, 2; see supplementary Table S1 and S2 of supplementary file 1). On the proboscides of studied moth species, we found the pollen grains of 21 plant families viz. Acanthaceae, Anacardiaceae, Apocynaceae, Asteraceae, Balsaminaceae, Betulaceae, Elaeocarpaceae, Ericaceae, Euphorbiaceae, Fabaceae, Fagaceae, Lamiaceae, Malvaceae, Myrtaceae, Oleaceae, Passifloraceae, Plantaginaceae, Poaceae, Rosaceae, Salicaceae and Verbenaceae. The pollen grains of Betulaceae are most frequently found (39.89% of total pollen grains found on proboscides of all PTMS), followed by Fabaceae (26.62%), Rosaceae (15.44%) and Ericaceae (7.66%). Further, we found the largest number of PTMS came from the Geometridae (53 species; 37.85%), followed by Erebidae (50 species; 35.71%), Crambidae and Noctuidae (14 species each; 10%), Drepanidae (7 species; 5%) and Nolidae (2 species, 1.42%). Composition of PPMS is similar, with highest number from Geometridae (36 species; 39.56%), followed by Erebidae (31 species; 34.06%), Noctuidae (11 species; 12.08%), Crambidae (8 species; 8.79%), Drepanidae (3 species; 3.29%) and Nolidae (2 species; 2.19%). Of the 91 PPMS, 60 species carried the pollen of a single plant family, and 23 species carried pollen grains of two plant families. Only six moth species i.e., *Hypocala* sp. (Erebidae: Arctiinae), *Callindra nyctemerata* (Erebidae: Arctiinae), *Achaea janata* (Erebidae: Erebinae), *Oxyodes scrobiculata* (Erebidae: Erebinae), *Thysanoplusia orichalcea* (Noctuidae: Plusiinae) and *Cyclidia substigmaria* (Drepanidae: Cyclidinae) carried pollen grains of three plant families and two moth species, *Ourapteryx* sp. (Geometridae: Ennominae) and *Sarbanissa catacoloides* (Noctuidae: Agaristinae) carried pollen of five plant families. *Teliphasa* sp. (Epipaschiinae: Crambidae) and *Cuculia* sp. (Noctuidae: Cuculliinae) are found to carry the highest quantity of pollen (more than 1000 pollen grains of Fabaceae and Betulaceae).

### Season-wise distribution of PTMS and PPMS at different altitudes in the Himalayan ecosystem

In our study, moths are found to carry pollen across the altitudinal gradient sampled up to 3000 m, however, pollen composition from different families varied at different altitude and in different seasons. The maximum number of PTMS are found in sub-tropical zone (1000–2000 m) (74 species; 52.85%), followed by temperate zone (2000–3000 m) (69 species; 49.28%), and in tropical zone (0–1000 m) (9 species; 6.42%). Drepanidae transported pollen in higher altitudes, beyond 2500 m.

#### Pre-monsoon season

**Figure 3:**
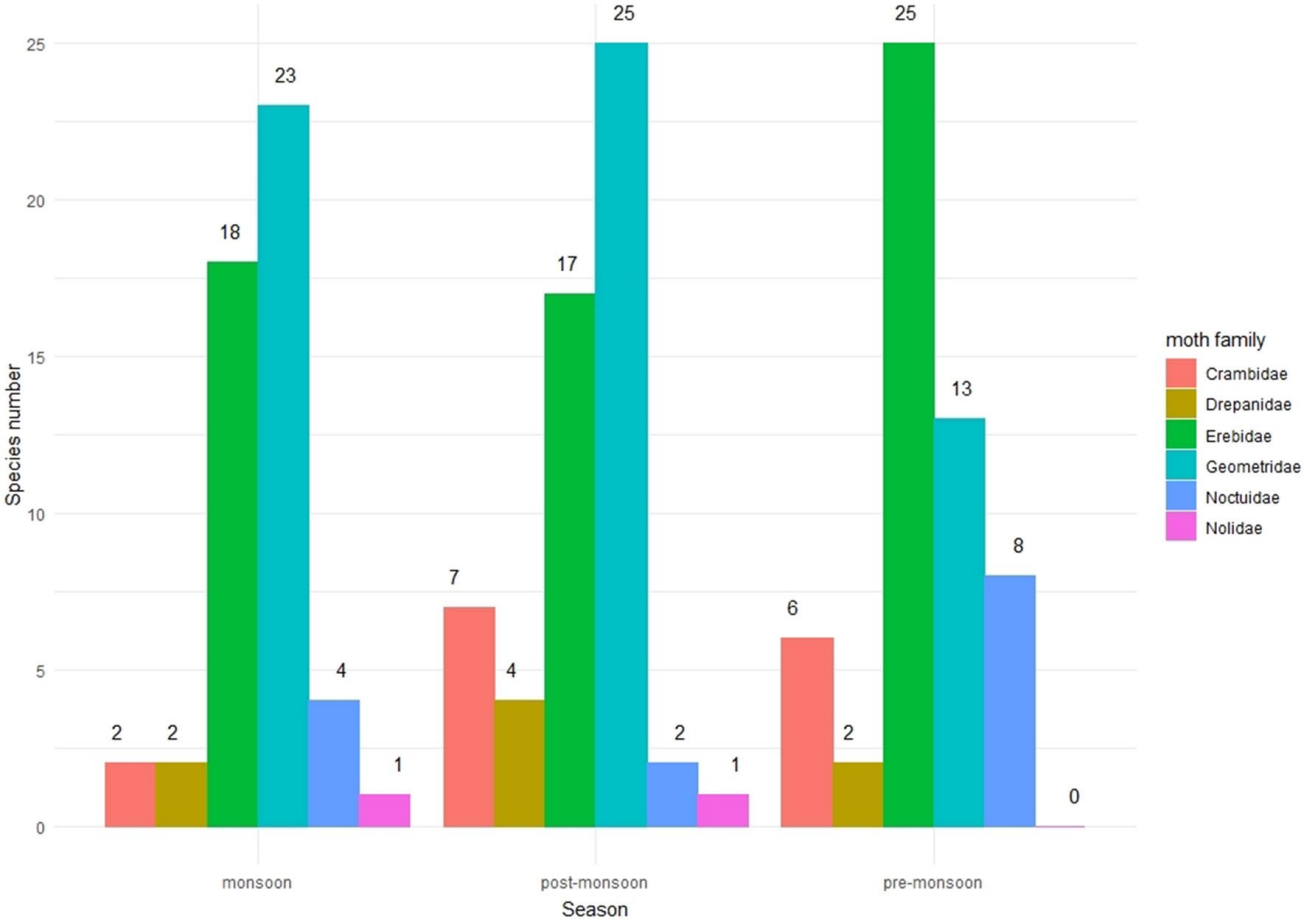
Season-wise species composition of PTMS.

In pre-monsoon, we found 54 and 40 PTMS and PPMS, respectively. 25 PTMS (46.29%) came from Erebidae, followed by Geometridae (13; 24.07%), Noctuidae (8; 14.81%), Crambidae (6; 11.11%) and Drepanidae (2; 3.70%) (Fig. 3; see supplementary Table S3 of supplementary file 1). No Nolidae are recorded from this season. During the pre-monsoon, moths carried the pollen of 15 plant families (Fabaceae, Betulaceae, Verbenaceae, Ericaceae, Balsaminaceae, Malvaceae, Poaceae, Rosaceae, Salicaceae, Elaeocarpaceae, Oleaceae, Asteraceae, Plantaginaceae, Fagaceae and Euphorbiaceae).

Altitudinally, in tropical zone (0–1000 m), a single PTMS, *Pelagodes* sp. (Geometridae) is reported. In sub-tropical zone (1000–2000 m), we found 23 PTMS, of which Erebidae dominated with 11 PTMS, followed by Geometridae (6 PTMS), Noctuidae (5 PTMS) and Drepanidae (1 PTMS). No PTMS are recorded in Crambidae and Nolidae. In this zone, *Cuculia* sp. (Erebidae) reported to transport maximum (more than 1000) pollen grains, followed by *Pericyma umbrina* (223) and *Abraxas* sp. (103). In sub-tropical zone, PTMS transported maximum pollen grains of Rosaceae, followed by Ericaceae, Fabaceae and Betulaceae, along with pollen of other 5 plant families. In temperate zone (2000–3000 m), we report 41 PTMS, with Erebidae having the highest PTMS (18), followed by Geometridae (12 PTMS), Crambidae (6 PTMS), Noctuidae (3 PTMS) and Drepanidae (2 PTMS). No PTMS are found in Nolidae (see supplementary file 2). In this zone, *Teliphasa* sp. (Crambidae) transported maximum pollen (more than 1000), followed by *Vamuna remelana* (200) and *Semiothisa* sp. (175). In temperate zone, PTMS transported the pollen of 14 plant families, with Fabaceae dominated the pollen composition.

#### Monsoon season

In monsoon season, we found 50 and 31 PTMS and PPMS, respectively. Of which, 23 PTMS (46%) are Geometridae, followed by Erebidae (18; 36%), Noctuidae (4; 8%), Crambidae and Drepanidae (2 each; 4%) and only one PTMS from Nolidae (Fig. 3; see supplementary Table S3 of supplementary file 1). We found the pollen of 9 different plant families (Rosaceae, Fabaceae, Betulaceae, Ericaceae Oleaceae, Malvaceae, Salicaceae, Poaceae and Passifloraceae), of which first four families dominated the pollen composition. Among the three seasons, this is the lowest number of plant families represented on the proboscides of moths.

Altitudinally, in tropical zone (0–1000 m), we report 9 PTMS transporting pollen of 3 plant families, Betulaceae, Fabaceae and Rosaceae. Among pollen transporting moths, Erebidae dominated with 4 PTMS (44% ). No PTMS are found in Nolidae and Drepanidae. In sub-tropical zone (1000–2000 m), 30 PTMS are reported to transport pollen of 8 plant families. Geometridae dominated with 14 PTMS, followed by Erebidae (11 PTMS). In this zone, *Sarcinodes aequilinearia* (Geometridae) transported maximum pollen, followed by *Percnia felinaria* (Geometridae), and *Siglophora ferreilutea* (Nolidae). No PTMS are reported from Crambidae. In sub-tropical zone, pollen grains of Rosaceae are transported maximum in numbers followed by Ericaceae, Fabaceae and Betulaceae. In temperate zone (2000–3000 m), 15 PTMS are found to carry pollen grains of 7 plant families. In this zone, Geometridae dominated with 7 PTMS, followed by Erebidae (6 PTMS). No PTMS are recorded in Nolidae and Drepanidae (see supplementary file 2). *Sarcinodes restitutaria* (Geometridae) transported highest pollen, followed by *Achaea janata* (Erebidae). In this zone, PTMS transported highest pollen of Rosaceae, followed by Betulaceae and Ericaceae.

#### Post-monsoon season

In post-monsoon season, of the 56 PTMS and 29 PPMS respectively, 25 PTMS (44.64%) are Geometridae, followed by Erebidae (17; 30.35%), Crambidae (7; 12.5%), Drepanidae (4; 7.14%), Noctuidae (2; 3.57%) and Nolidae (1; 1.78%) (Fig. 3; see supplementary Table S3 of supplementary file 1). During the season, moths carried the pollen of 16 plant families, of which, pollen of Euphorbiaceae, Salicaceae, Acanthaceae, Anacardiaceae, and Lamiaceae are very few.

Altitudinally, in post-monsoon, no PTMS are reported from tropical zone (0–1000 m). In sub-tropical zone (1000–2000 m), 32 PTMS are reported to carry pollen of 14 plant families. Geometridae dominated with 23 PTMS, followed by Erebidae (7 PTMS). In this zone, *Dindica olivacea* (Geometridae) transported maximum pollen, followed by *Ectropis* sp. (Geometridae), and *Cyana bellissima* (Erebidae). No PTMS are recorded from Crambidae, Nolidae and Drepanidae. Pollen of Betulaceae are transported in maximum, followed by Fabaceae, Poaceae and Myrtaceae. In temperate zone (2000–3000 m), we found 15 PTMS transporting pollen of 9 plant families, with Geometridae dominating the PTMS composition (7 PTMS), followed by Erebidae (6 PTMS). In this zone, major PTMS are *Tyana* sp. (Nolidae), and *Hesudra divisa* (Erebidae). No PTMS are recorded from Drepanidae (see supplementary file 2). PTMS are found to carry maximum pollen of Betulaceae followed by Malvaceae and Fabaceae.

### Pollen transfer networks

**Figure 4:**
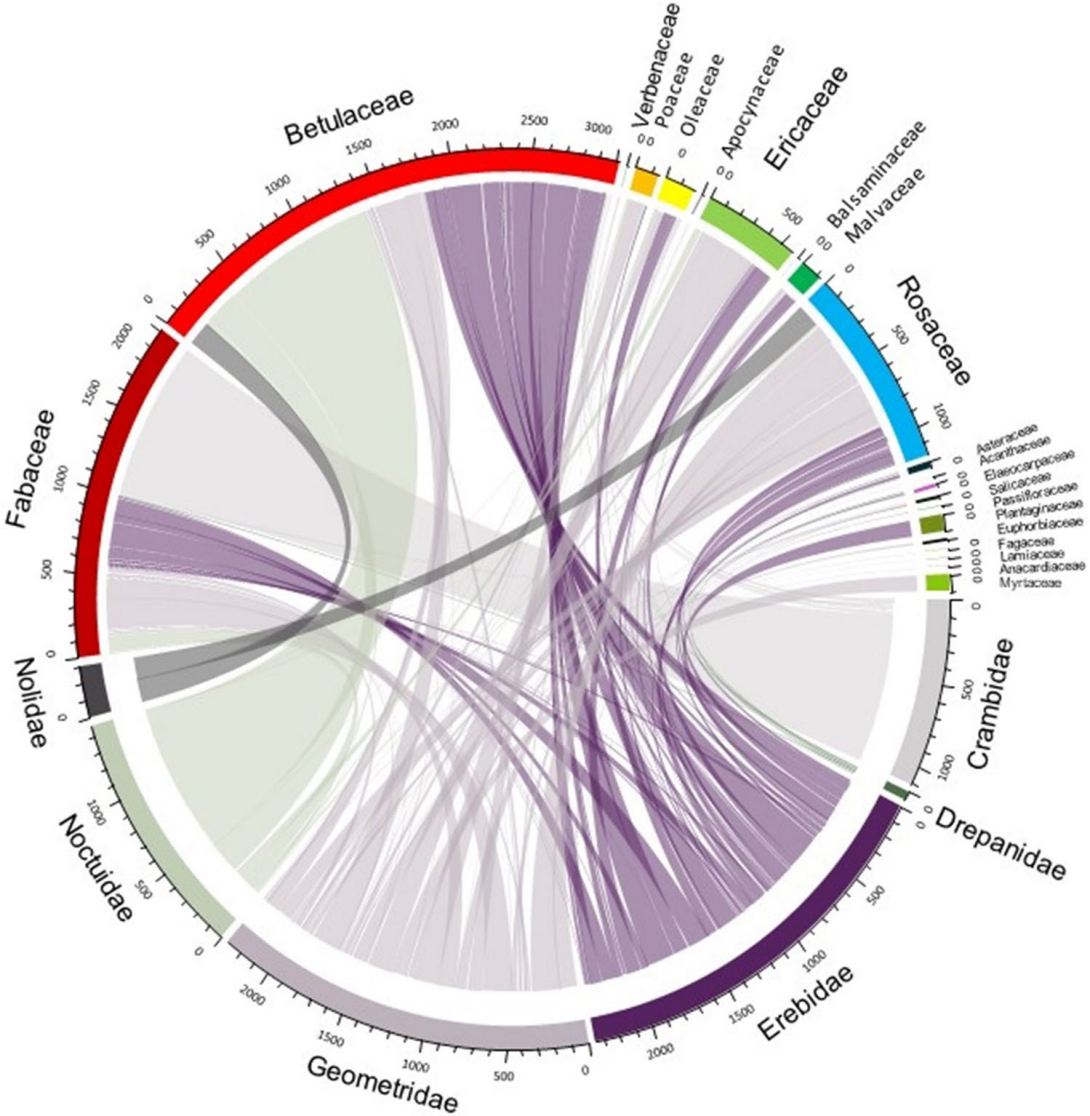
Circos plot to visualise the interaction of moth families with the plant families (based upon supplementary file 2).

**Figure 5:**
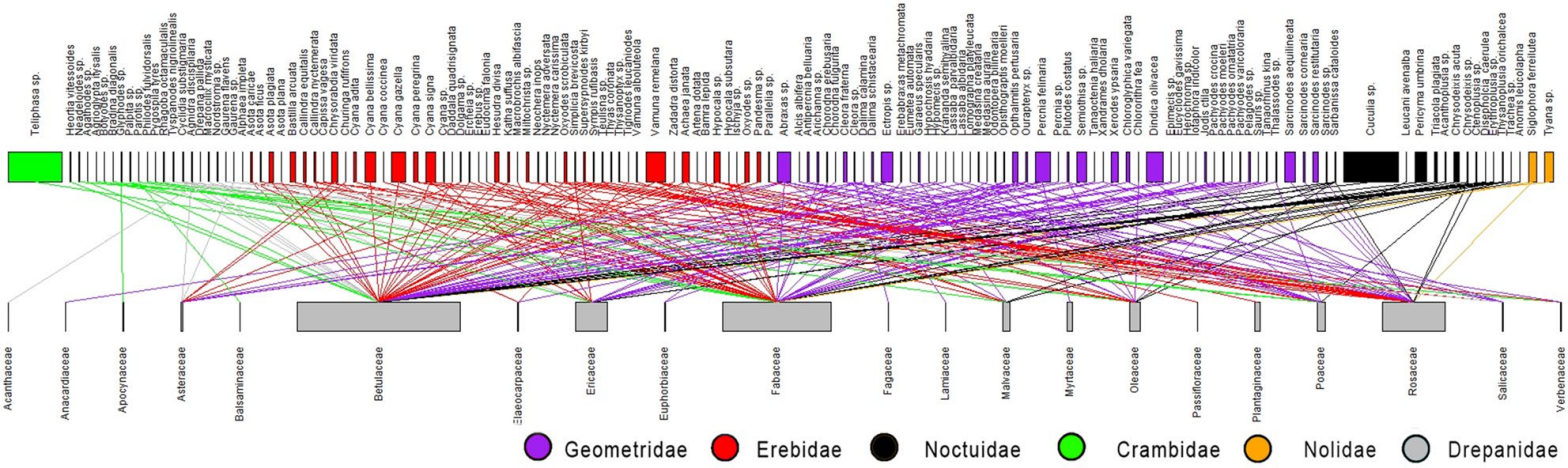
Consolidated settling moth-plant pollen transfer network (width of higher-level boxes indicates number of pollen grain carried by moths whereas width of lower level boxes indicate total pollen grain of a plant family transported).

**Figure 6:**
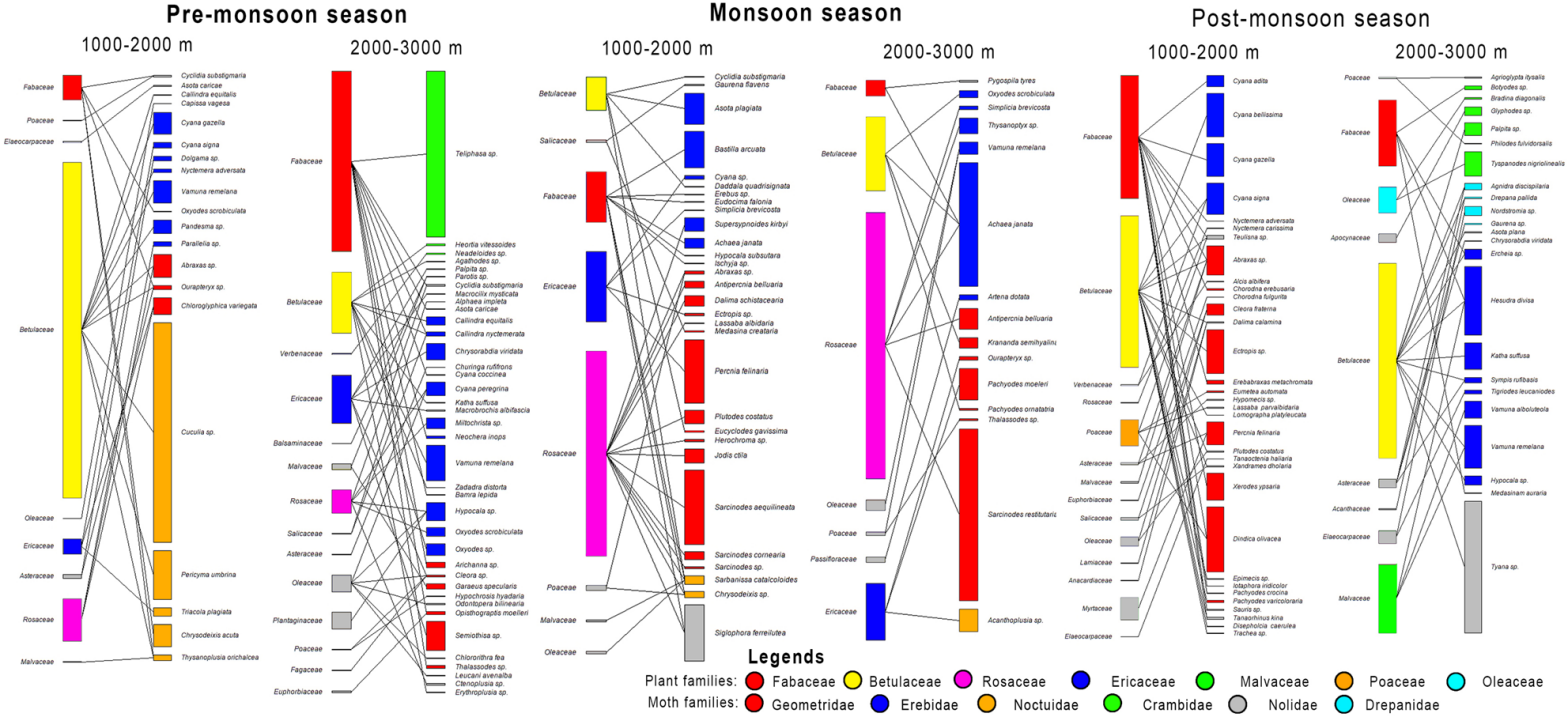
Seasonal and altitudinal wise settling moth-plant pollen transfer network (dominating plant families and moth families are coloured).

The settling moth-plant pollen transfer network comprises 140 moth species and 21 plant families (Figs. 4, 5; Based upon supplementary file 2; the script for the bipartite network construction is given as supplementary file 3). In the first network (Fig. 5) we present the consolidated data (for all seasons and all altitudes) and in the second network (Fig. 6) the data is analysed seasonally and altitudinally. The consolidated network has 1.31 links per species with a linkage density of 5.91 and 0.48 as the interaction evenness. The interaction richness is found to be 3.90. The network shows the high degree of selectivity (H_2_ʹ = 0.86) with nested interaction (nestedness = 5.41). All the calculated network matrices are provided in Table 1. The PDI score (supplementary file 4) indicate that 9 plant families viz. Acanthaceae, Anacardiaceae, Apocynaceae, Balsaminaceae, Fagaceae, Lamiaceae, Myrtaceae, Passifloraceae and Plantaginaceae show complete specialism in relation with moths. Similarly in case of moths we found that 98 settling moth species exhibit specialism and rest are generalists .

**Table 1:** Seasonal and altitudinal wise network-level matrices of settling moth-plant pollen transfer networks.

Network-level matrices	Consolidated	Monsoon (sub-tropical)	Monsoon (temperate)	Pre-monsoon (sub-tropical)	Pre-monsoon (temperate)	Post-monsoon (sub-tropical)	Post-monsoon (temperate)
Connectance	0.071	0.16	0.17	0.15	0.09	0.1	0.14
Nestedness	5.41	19.02	31.12	20.28	12.43	10.61	25.64
H_2_ʹ	0.86	0.96	0.86	0.98	0.98	0.86	0.89
Links per species	1.31	1	0.82	0.93	0.94	1	0.94
Interaction evenness	0.48	0.47	0.45	0.36	0.35	0.44	0.45
Shannon diversity	3.90	2.55	2.07	1.88	2.21	2.68	2.38
Interaction strength asymmetry	0.50	0.5	0.42	0.39	0.53	0.32	0.4
Linkage density	5.91	2.74	2	2.04	1.6	2.75	1.94

### Season-wise and altitudinal zone-wise pollen transfer network of settling moths

The season-wise and altitudinal zone-wise settling moth-plant pollen transfer network is constructed to get the information on variation in pollen transfer networks in various seasons across different altitudinal zones in Himalayan ecosystem of North-East India (Fig. 6 based upon supplementary file 2; for network matrices see supplementary file 4). Due to lesser number of PTMS as well as plant families, the matrices for data of tropical zone are not analysed.

#### Pre-monsoon season

During this season the bipartite moth-plant pollen transfer network in sub-tropical zone (1000–2000 m) shows higher values of connectance (0.15), nestedness (20.28), interactive evenness (0.36) and linkage density (2.04), whereas, in temperate zone (2000–3000 m), the network shows higher values of links per species (0.94), Shannon’s diversity (2.21), and interaction strength asymmetry (0.53). The PDI score indicate that the number of specialist and generalist moth species, and the number of specialist and generalist plant families are higher in temperate zone. Interestingly the selectivity (H_2_’) remained same for both zones i.e., 0.98 (Table 1; Based upon supplementary file 2; see supplementary file 4).

#### Monsoon season

In monsoon, the bipartite network in sub-tropical zone (1000–2000 m) shows higher values of interactive evenness (0.47), linkage density (2.74), links per species (1), and Shannon’s diversity (2.55) whereas, in temperate zone (2000–3000 m), the network shows higher values of connectance (0.17), nestedness (31.12) (Table 1). The PDI score indicate that the number of specialist and generalist moth species and generalist plant families is higher in sub-tropical zone (see supplementary file 4). Selectivity (0.96) is higher in sub-tropical zone.

#### Post-monsoon season

In post-monsoon season, values of the network matrices viz. links per species (1), Shannon’s diversity (2.68) and linkage density (2.75) are higher in sub-tropical zone (1000–2000 m) whereas, the values of connectance (0.14), nestedness (25.64), interaction evenness (0.45) and interaction strength asymmetry (0.40) are higher in temperate zone (2000–3000 m). The number of specialist moth species, specialist plant families, generalist moth species are higher in sub-tropical zone but the number of generalist plant families remain same for both sub-tropical and temperate zone. Selectivity (H_2_ʹ = 0.89) is higher in temperate zone (Table 1 based upon supplementary file 2; see supplementary file 4).

## Discussion

Our results are based on one of the largest data sets (140 species) interpreting the role of settling moths in pollination and is the first of its kind to understand the settling moths-plant pollination interactions in the central and eastern Himalayan ecosystem of India. Our approach of using the proboscis by isolating from the moth head and relaxing it for pollen extraction, provides a more robust assessment of the pollen carrying capacity of the moths, because (1) by using the proboscis for the pollen collection, the likelihood of contamination due to pollen rain is reduced (as swabbing of other parts of body result in collection of extra pollen grains from the body, unrelated to flower visitation), and (2) de-coiling the proboscis enables the study of pollen grains stuck within the coiling of the proboscis (which may remain there during swabbing).

We found that substantial proportion (about 65%) of PTMS carried sufficient quantities of pollen grains (five or more than five) to be considered as potential pollinators (PPMS). Geometridae and Erebidae turned out to be the most important for the pollen transportation in the Himalayan region and on the other hand, pollen grains of Betulaceae, predominantly a wind-pollinated plant family, are transported in maximum. In our analysis, we include the Betulaceae related data as some wind-pollinated plant families may benefit from enhanced dispersal by insects, although the extent of this relationship is unknown^45^. Some recent studies reveal that some of the insect pollinators, particularly bees and syrphid fly species, collect pollen from a broad range of wind-pollinated plant species^46–48^, and also, some other studies^49–57^ identified insect pollination in plant species which were presumed to be wind-pollinated. Our study may open some new horizons in the field of plant-pollinator interactions, a field with large gaps in knowledge. The present study reveals that the season and altitude affect the role of moths as PTMS greatly. In sub-tropical zone (1000–2000 m), we found highest number of PTMS as well as the diversity of pollen grains in post-monsoon whereas in temperate zone, the maximum diversity of PTMS and pollen grain is reported in pre-monsoon. The number of PTMS, PPMS and the diversity of pollen grains are lowest in the monsoon season. This may be due to the heavy rainfall with tropical rainstorms restricting the moth activities, as the weather parametres like rainfall, daily temperature plays a significant role in moth abundance, richness in a particular habitat^58^. Overall, we found maximum diversity of PTMS from higher sub-tropical zone (1500–2000 m) (44 PTMS) to lower temperate zone (2000–2500 m) (65 PTMS), a Himalayan zone with high floral diversity^59–61^. The tropical zone of Himalaya is mainly dominated with plant families like Fabaceae, Betulaceae, Euphorbiaceae, Theaceae and Asteraceae^61^, among which most of the species are trees. Whereas, in sub-tropical and temperate zone the floral component is characterized by plant families like Elaeocarpaceae, Oleaceae, Solanaceae, Rubiaceae, Zingiberaceae, Verbenaceae, Ericaceae, Asteraceae, Rosaceae and Orchidaceae^59,60,62,63^ with dominance of shrubs and herbs. In our study, higher proportion of pollen from four plant families i.e., Fabaceae, Betulaceae, Ericaceae and Rosaceae is a possible indication of high preferences of settling moths towards these plant families, mainly for nectaring or may be for some non-nectar resource. Some non-lepidopteran pollinators visit wind-pollinated plants to collect or feed on pollen, and for non-floral resources i.e. insect honey dew, plant resins or resinous secretions^45,64–67^ but our understanding about the attraction of settling moths towards small flowers with a very low quantity of nectar is very limited and requires further exploration.

We constructed the pollen number based network following Banza et al.^68^ (with selectivity value of 0.79), and our network matrices also revealed high degree of selectivity (0.86) of settling moths in Himalayan ecosystem, which is highest value as compared to the other studies conducted on nocturnal moths in several ecosystems around the world^12,68–70^, thus, showing the importance of our study conducted in a larger spatial scale. The PDI score revealed that major proportion (98 species; 70%) of PTMS are specialist and only 42 species (30%) are generalists. High degree of specialism in moths is a strong indication of inter-dependency, and there are high possibilities that the dwindling moth diversity will adversely affect the plant diversity, and the other way round also. In our consolidated network, the value of interaction strength asymmetry (0.50) indicates that the specialised species are interacting with the generalised species and the vice versa.

We found significant correlation between the polyphagous nature of *Achaea janata* (Erebidae: Erebinae) and its ability to transfer pollen of different plant families. *A. janata*, commonly known as Castor oil semi-looper or Croton caterpillar, is a widely distributed species in the region and more broadly, it is a well-known pest of various economically important plants like *Arachis hypogaea* (Fabaceae), *Rosa chinensis* (Rosaceae), *Tamarindus indica* (Fabaceae), *Glycine max* (Fabaceae), *Vigna mungo* (Fabaceae), and *Dalbergia sisso* (Fabaceae)^71^. However, our results establish *A. janata* as a potential pollinator of three plant families, an addition to the knowledge provided by few such studies^72^ indicating that the moths can provide net benefits as pollinators even when acting as larval herbivores of the same species. The phenomenon revealed that the species-interactions are much more complicated than we think of.

We believe that our results will lay a strong foundation for the studies related to moth-plant interaction, particularly in the Indian context and will further strengthen the concepts of ecosystem conservation rather than concentrating on few taxa. The present dataset is a tip of an iceberg as there are about 12,000 moth species in India^73^ and about 160,000 in the world^74^ and their abundance is still unknown to the science. We are quite sure that data generation and accumulation of knowledge through such type of studies will definitely strengthen our understanding about the role of nocturnal insect pollinators and will help in a better way regarding the management decisions for conservation, biodiversity, and agriculture.

## Methods

### Moth collection and sampling design

**Figure 7:**
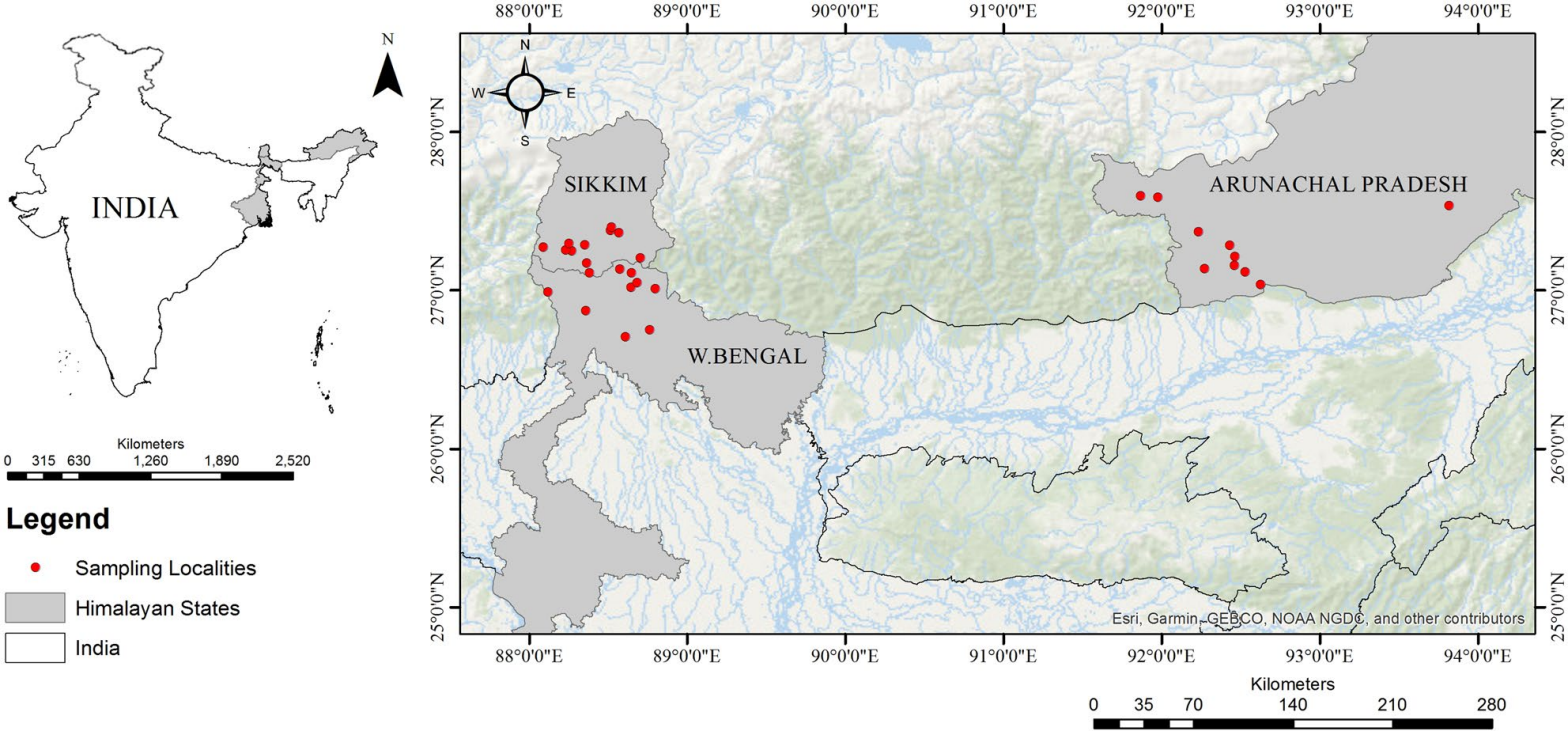
Localities sampled in this study (ArcGIS version 10.5, URL: https://www.arcgis.com/index.html).

Representatives of settling moth families were collected from September, 2018 to October, 2019 in three different seasons i.e., pre-monsoon (May–June), monsoon (July–September) and post-monsoon season (October–November), using vertical sheet light traps operating with 160-W mercury vapour lamps from 24 randomly chosen sampling sites of Arunachal Pradesh, North Bengal and Sikkim (Fig. 7; see Table S4 of supplementary file 1). For collection, a total of 7 surveys were conducted. Each locality was surveyed for two days per season. Light trapping was done from 6PM to 6AM. The moths were collected individually in the jars euthanized with ethyl acetate. A maximum of 2–3 specimens of each species were collected. Moths were processed and preserved as per standard techniques in Lepidopterology^75^.

The three clearly distinct seasons of Himalayan region were sampled as they greatly affect the floral and faunal components of the area under study^76^. The collections were made from a very large altitudinal gradient covering up to 3000 m and due to which, we interpreted our data in consolidate, as well as seasonally (pre-monsoon, monsoon and post-monsoon) and altitudinally (tropical zone (0–1000 m), sub-tropical zone (1000–2000 m) and temperate zone (2000–3000 m)).

### Isolation of moth proboscis and light microscopy of Fuchsin jelly slides

We used proboscides (c.a. 1800) to investigate the pollen loads (not the other parts of the body like head or legs) and also, for the first time, we separated the proboscis of each moth and directly processed it for the study of pollen (rather than swabbing). Our method reduced the chances of contamination due to pollen rain and it also enabled the study of those particular pollen grains which are found within the coiling of the proboscis, which may remain unstudied during swabbing. We isolated the proboscis from each moth using sterilised forceps. Each proboscis was kept in a separate vial with code number. To relax, the proboscis of each moth was placed on a glass slide and treated with few drops of 1:1 ratio of phenol and glycerol solution for 1–2 min. Afterwards, one to two drops of 50% solution of basic Fuchsin dye were added and the slide was mounted with cover slips and sealed with nail varnish. A Nikon 50i microscope with DP–25 digital camera (40 X magnification) was used to photograph the slides and to count the pollen grains.

### SEM analysis of isolated proboscis and pollen

The isolated proboscides of some specimens were also scanned with SEM, and photographs of pollen were taken using ZEISS Evo18 image acquiring software v5.09.

### Moth and pollen grain identification

Moths were identified using various available literature, publications^77–82^ and websites viz. www.mothsofindia.org^83^, www.inaturalist.org^84^. The classification followed here is given by Nieukerken^85^. Pollen grains were identified from palynological literature, books and websites including: www.paldat.org^86^, www.globalpollenproject.org^87–91^.

### Pollen transfer network construction and calculation of network matrices

Seven matrices (one consolidated, and two separate matrices (for sub-tropical and temperate zone) for each season) (see supplementary file 2) are prepared by keeping all the settling moth species along the rows and the plant families along the columns. The number of pollen grains from each plant family found on individual moth species is taken as the value for constructing the mutualistic network^44^. For quantification of all the mutualistic network parametres ‘bipartite’ package of RStudio is used^70^. The network-level metrics are (1) connectance (reflects the possible recorded links in the network or standardised number of species combinations)^92^, higher value indicates more connectance, (2) nestedness (range 0–100, higher value reflects more complexity of the network)^93^, (3) specialization or H_2_' (range 0–1, higher value indicates more specialist species are interacting against the generalist species)^93,94^, (4) links per species (quantify links of species present in a mutualistic network), (5) interaction evenness (evenness of interactions of animals in a network based on Shannon’s diversity, higher value reflects more evenness)^95^, (6) Shannon’s diversity (to calculate the interaction richness of the network, high value indicates higher diversity), (7) interaction strength asymmetry (quantify weather the specialised species are interacting with generalised species in other level or vice versa)^96^, (8) linkage density (measures the density of linkage in a network)^97^ and (9) species-level paired difference index (PDI) (measured to show the specialization of individual species for commonly visited plant family)^98^. The PDI score ranges from 0 to 1, 0 indicate complete generalism and 1 indicate total specialism in the plants’ relationship with moths.

## Supplementary Information

Supplementary Information 1.

Supplementary Information 2.

Supplementary Information 3.

Supplementary Information 4.
