# Supplementary material for: Settling moths are the vital component of pollination in Himalayan ecosystem of North-East India, pollen transfer network approach revealed
Source: Sci Rep. 2022 Feb 17;12:2716. doi: 10.1038/s41598-022-06635-4 (PMC8854426; doi:10.1038/s41598-022-06635-4)
Supplement: Supplementary file 1 — Supplementary Information 1. [file 41598_2022_6635_MOESM1_ESM.docx]

**Table S1** Ten major plant families and their pollen load found on proboscides of PTMS

| **Plant family** | **Total number of pollen grains** |
| --- | --- |
| Asteraceae | 40 |
| Betulaceae | 3022 |
| Ericaceae | 580 |
| Fabaceae | 2017 |
| Malvaceae | 129 |
| Myrtaceae | 100 |
| Oleaceae | 190 |
| Plantaginaceae | 100 |
| Poaceae | 142 |
| Rosaceae | 1170 |

**Table S2** Family-wise species composition of PTMS and PPMS

| **Moth Family** | **Number of PTMS** | **Number of PPMS** |
| --- | --- | --- |
| Geometridae | 53 | 36 |
| Erebidae | 50 | 31 |
| Noctuidae | 14 | 11 |
| Crambidae | 14 | 8 |
| Drepanidae | 7 | 3 |
| Nolidae | 2 | 2 |
| Total | 140 | 91 |

**Table S3** Season-wise altitudinal PTMS composition

| **Moth family** | **Season** | | |
| --- | --- | --- | --- |
|  | **Pre-monsoon** | **Monsoon** | **Post-monsoon** |
| Crambidae | 6 | 2 | 7 |
| Drepanidae | 2 | 2 | 4 |
| Erebidae | 25 | 18 | 17 |
| Geometridae | 13 | 23 | 25 |
| Noctuidae | 8 | 4 | 2 |
| Nolidae | 0 | 1 | 1 |

**Table S4** Details of the localities sampled during this study

| Sl. No. | State | Survey locality | Latitude (°) | Longitude (°) | Elevation (in m) |
| --- | --- | --- | --- | --- | --- |
| 1 | Arunachal Pradesh | Charrit | N27.2128 | E92.4641 | 1429 |
| 2 |  | Shergaon | N27.1341 | E92.2738 | 2044 |
| 3 |  | Lama camp | N27.1575 | E92.4608 | 2330 |
| 4 |  | Jang | N27.5876 | E91.9789 | 2151 |
| 5 |  | Salari | N27.3288 | E92.4502 | 2116 |
| 6 |  | Ziro | N27.5347 | E93.8205 | 1588 |
| 7 |  | Old ziro | N27.5965 | E93.8250 | 1582 |
| 8 |  | Helipad | N27.2102 | E92.5083 | 2316 |
| 9 | Northern West Bengal | Rishikhola | N27.1632 | E88.6453 | 575 |
| 10 |  | Zhandi | N27.0188 | E88.6472 | 1742 |
| 11 |  | Rishop | N27.1086 | E88.6486 | 2156 |
| 12 |  | Eche Gaon | N27.1336 | E88.5744 | 1773 |
| 13 |  | Charkhole | N27.0001 | E88.5340 | 1557 |
| 14 |  | Kuwapani | N27.0462 | E88.6843 | 1531 |
| 15 |  | Danagaon | N27.0691 | E88.2730 | 1795 |
| 16 |  | Sitong | N26.8705 | E88.3605 | 328 |
| 17 |  | Jayanti | N26.7038 | E88.6094 | 101 |
| 18 | Sikkim | Panthyang | N27.3639 | E88.5686 | 2022 |
| 19 |  | Namchi | N27.1695 | E88.3658 | 1637 |
| 20 |  | Ravangla | N27.2857 | E88.3540 | 2013 |
| 21 |  | Tintek | N27.3766 | E88.5154 | 1496 |
| 22 |  | Bermiak | N27.2532 | E88.2332 | 1453 |
| 23 |  | Geyzing | N27.2950 | E88.2534 | 1622 |
| 24 |  | Dikchu | N27.3989 | E88.5232 | 794 |
